# Baicalin suppresses SVCV replication and is associated with modulation of the AMPK/mTOR-mediated autophagy pathway

**DOI:** 10.3389/fimmu.2026.1853770

**Published:** 2026-08-20

**Authors:** Mingyang Xue, Mengwei Zhang, Yang Hu, Chen Xu, Yan Meng, Yuding Fan, Shaohui Zhou, Lei Liu, Weiguang Kong, Yong Zhou

**Affiliations:** 1 Yangtze River Fisheries Research Institute, Chinese Academy of Fishery Sciences, Wuhan, China; 2 College of Fisheries, Huazhong Agricultural University, Wuhan, Hubei, China; 3 State Key Laboratory for Managing Biotic and Chemical Threats to the Quality and Safety of Agro-Products, Ningbo University, Ningbo, China; 4 Hunan Zhangjiajie Giant Salamander National Nature Reserve Affairs Center, Zhangjiajie, China; 5 Institute of Hydrobiology, Chinese Academy of Sciences, Wuhan, China

**Keywords:** AMPK/mTOR, antiviral, autophagy, baicalin, spring viremia of carp virus

## Abstract

Spring viremia of carp virus (SVCV) poses a major threat to carp aquaculture due to the lack of effective antiviral strategies. This study investigates the antiviral potential of baicalin in epithelioma papulosum cyprini (EPC) cells and zebrafish. Baicalin exhibits antiviral activity during both pretreatment and post-infection treatment and shows direct virucidal activity during virus–compound co-incubation. Moreover, baicalin alleviated oxidative stress, preserved mitochondrial function, and reduced apoptosis in infected cells. Proteomic analyses revealed that baicalin suppressed SVCV replication by modulating the AMPK/mTOR signaling pathway, as evidenced by decreased AMPK phosphorylation, increased mTOR phosphorylation, and subsequent inhibition of autophagy. These findings demonstrate that baicalin significantly suppresses SVCV replication and is associated with modulation of the AMPK/mTOR-mediated autophagy pathway. These findings identify baicalin as a promising antiviral candidate against SVCV and provide a foundation for further evaluation in economically important aquaculture species.

## 1 Introduction

Spring viremia of carp virus (SVCV) is a negative-strand RNA virus that belongs to the family Rhabdoviridae. This pathogen is known to cause significant mortality in common carp (*Cyprinus carpio*) and other species within the *Cyprinidae* family (1). SVCV exploits the host’s antiviral responses, such as apoptosis and cellular autophagy, to facilitate its own replication. This process results in mitochondrial dysfunction, increased accumulation of intracellular reactive oxygen species (ROS), and decreased expression of anti-inflammatory proteins. Consequently, the virus induces host oxidative stress, which enhances viral invasion and release (2–5). Ongoing investigations into the complexities of SVCV’s pathogenic pathways are uncovering promising avenues for identifying novel therapeutic targets and developing effective antiviral strategies.

The increasing recognition of naturally derived chemical substances with antiviral properties represents a significant shift in pharmaceutical development, providing a rich source of innovative therapeutic options (6). Among these substances, flavonoids—polyphenolic compounds produced through plant metabolism—are particularly notable due to their diverse biological activities and low toxicity. Baicalin, a natural flavonoid found in the roots of *Scutellaria baicalensis* Georgi, has been shown to possess antioxidant properties, boost immunity, and inhibit viral activity (7). Previous research has demonstrated that baicalin significantly inhibits the replication of viruses such as influenza A virus (IAV) and respiratory syncytial virus (RSV) (8–10). However, its use in combating aquaculture viruses remains limited, and the mechanisms underlying its antiviral effects are largely unexplored.

Virus-induced oxidative stress is closely associated with the activation of mitochondrial apoptosis, a critical host response that can influence both viral replication and disease progression (11). Excessive reactive oxygen species (ROS) promote mitochondrial dysfunction and activate pro-apoptotic members of the Bcl-2 family, including Bad and Bax, leading to caspase activation and apoptotic cell death. Increasing evidence also indicates extensive crosstalk between apoptosis and autophagy during viral infection, and modulation of these pathways has emerged as a promising strategy for limiting virus-induced cellular damage and improving antiviral efficacy (12).

Besides apoptosis, autophagy is an evolutionarily conserved intracellular degradation process that functions as an intrinsic host defense mechanism by eliminating misfolded proteins, damaged organelles, and invading pathogens (13). Autophagic flux refers to the complete dynamic process from autophagosome formation to lysosomal degradation, which is tightly regulated by multiple autophagy-related proteins and signaling pathways (14). Among these, the mammalian target of rapamycin (mTOR) and adenosine monophosphate-activated protein kinase (AMPK) signaling pathways coordinately regulate the activation of UNC-51-like kinase 1 (ULK1), thereby controlling the initiation of autophagy (15). During autophagosome formation, LC3-I is lipidated to form LC3-II through the coordinated actions of ATG7 and ATG3, and LC3-II is widely recognized as a hallmark of autophagosome formation (16, 17). Increasing evidence indicates that both DNA and RNA viruses can manipulate autophagic flux to promote viral replication, evade host immune surveillance, or establish persistent infection. Conversely, activation of autophagic flux can facilitate viral clearance depending on the specific virus–host interaction. Notably, several RNA viruses, including members of the family Rhabdoviridae, have evolved strategies to modulate autophagy for successful infection. Therefore, modulation of autophagy, particularly through the AMPK/mTOR signaling pathway, has emerged as a promising therapeutic strategy for antiviral intervention (18).

The current investigation examined the antiviral efficacy of baicalin across various treatment modalities in EPC cells and zebrafish, providing a basis for future evaluation in production environments. Furthermore, utilizing phosphoproteomics, we performed a comprehensive analysis of differentially expressed proteins, providing an enhanced understanding of the antiviral mechanisms of baicalin.

## 2 Materials and methods

### 2.1 Baicalin, cell, virus, and fish

Baicalin (CAS No: 21967-41-9, purity: 97.6%) was obtained from Shanghai YuanYe Bio-Technology Co., Ltd. It was dissolved in dimethyl sulfoxide (DMSO; Sigma-Aldrich, USA) to prepare a stock solution at a concentration of 50 mg/mL, which was stored at -20°C. Before each experiment, the stock solution was diluted with M199 medium containing 5% fetal bovine serum (FBS; Every Green, China) to achieve working concentrations ranging from 10 to 100 mg/L.

The EPC cell line was sourced from the China Typical Culture Collection Center, Wuhan University. EPC cells were cultured in Medium 199 (M199; Hyclone, USA) supplemented with 10% FBS at 25°C. The SVCV strain was isolated, identified, and preserved by the Fish Disease Research Laboratory at the Yangtze River Fisheries Research Institute, Chinese Academy of Fishery Sciences. Once confirmed, the strain was stored at ultra-low temperature (-80°C). The virus was propagated in EPC cells, and the viral suspension was quantified using the Reed-Muench method to determine viral titers.

Zebrafish (*Danio rerio*) used in this study were obtained from the National Zebrafish Resource Center at the Institute of Hydrobiology, Chinese Academy of Sciences. They were maintained at 28.0 ± 1.0°C, pH 7.5 ± 0.4, and fed twice daily at fixed intervals. One week before experimentation, the zebrafish were acclimatized to 18°C. All animal experiments were conducted following ethical standards and approved by the Animal Experimental Ethics Inspection of the Freshwater Fisheries Research Center, Chinese Academy of Fishery Sciences (YFI 2024-Zhouyong-0122). Before experimental procedures requiring handling, zebrafish were anesthetized by immersion in MS-222 (tricaine methanesulfonate; Sigma, USA) at a concentration of 100 mg/L. At the end of the experiments, following tissue collection, zebrafish were euthanized by immersion in an overdose of MS-222 (250 mg/L; Sigma, USA). Death was confirmed by the cessation of opercular movements and the absence of a response to external stimuli.

### 2.2 The cytotoxic effects of baicalin on EPC cells

Cell cytotoxicity was evaluated using the Cell Counting Kit-8 (CCK-8; Beyotime, China). EPC cells were seeded at a density of 1×10^4^ cells per well in 100 μL of culture medium in 96-well plates and incubated at 25°C for 16 to 24 h. Upon reaching 80% to 90% confluency, 100 μL of culture medium containing varying concentrations of baicalin was added to each well. Three parallel wells were maintained for each concentration. The cells were then cultured for an additional 72 h. Subsequently, 10 μL of CCK-8 solution was added to each well, followed by a 3 h incubation in the dark at 25°C. Absorbance at 450 nm was measured using a microplate reader to determine the cytotoxicity of baicalin on EPC cells compared to the MOCK group (DMSO content of 0.5%).

### 2.3 Detection of antiviral activity of baicalin on EPC cells

To analyze the *in vitro* antiviral activity of baicalin against SVCV, EPC cells infected with SVCV were treated with various concentrations of baicalin. Cell viability, expression levels of SVCV-G and SVCV-N protein mRNA, and viral titers were then assessed to determine the antiviral effects. EPC cells were initially cultured in 96-well, 12-well, and 6-well cell culture plates for 16 to 24 h. After this incubation period, the cells were exposed to virus dilutions containing 1 × 10^3^ TCID_50_/mL for 2 h. Subsequently, different concentrations of baicalin were added to the medium, and the cells were incubated for an additional 48 h. Cell viability was determined using the CCK-8 assay. Viral gene expression levels were measured using RT-qPCR, while viral titers were assessed using the Reed-Muench method to calculate the 50% tissue culture infective dose (TCID_50_).

### 2.4 Antiviral activity of baicalin under different treatment conditions in EPC cells

Pretreatment: EPC cells were pretreated with 63 mg/L baicalin for varying durations: 0 h, 6 h, 12 h, 24 h, 36 h, 48 h, and 72 h. After removing the supernatant, the cells were exposed to SVCV at a concentration of 1×10³ TCID_50_/mL for 48 h. Subsequently, cell samples were collected for the analysis of SVCV-G gene expression via RT-qPCR.

Co-incubation: EPC cells were co-incubated with 63 mg/L baicalin, with or without 1×10³ TCID_50_/mL SVCV, at 25°C for 15 min, 30 min, and 60 min. After a 48 h infection period, cell samples were collected, and SVCV-G gene expression was assessed using RT-qPCR.

Post-treatment: Following a 24 h SVCV infection period in EPC cells, 63 mg/L baicalin was added. The cells were then incubated at 25°C for an additional 24 h. Cell samples were subsequently collected, and the expression of the SVCV-G gene was quantified using RT-qPCR.

### 2.5 Cellular ultrastructure observation

After culturing EPC cells in a 6-well plate for 16 to 24 h, the cells were exposed to a virus dilution containing 1×10^3^ TCID_50_/mL for 2 h. Subsequently, the medium was replaced with fresh cell culture medium supplemented with 63 mg/L baicalin, and the cells were incubated for an additional 48 h. The cells were then fixed overnight in 2.5% glutaraldehyde. Surface and intracellular structures of the cells were examined using scanning electron microscopy (SEM; Hitachi, Japan) and transmission electron microscopy (TEM; Hitachi, Japan), respectively.

### 2.6 Baicalin’s influence on SVCV-induced cell apoptosis

EPC cells were seeded onto 20 mm circular coverslips (Biosharp, China) and into 12-well cell culture plates, both subjected to the same treatment regimen. Nuclear damage was visualized using confocal microscopy (Olympus, Japan); nuclei were stained with 1 mg/L 4’,6-diamidino-2-phenylindole (DAPI; Beyotime, China), and cell membranes were stained with 5 mg/mL 1,1’-dioctadecyl-3,3,3’,3’-tetramethylindocarbocyanine perchlorate (Dil; Beyotime, China). 5,5′,6,6′-Tetrachloro-1,1′,3,3′-tetraethyl-imidacarbocyanine iodide (JC-1; Beyotime, China) was used to assess mitochondrial membrane potential, a well-established fluorescent probe for this purpose. In healthy, polarized mitochondria, JC-1 forms aggregates, emitting red fluorescence. Conversely, in depolarized or unhealthy mitochondria, JC-1 remains in its monomeric form, emitting green fluorescence. Thus, shifts in mitochondrial membrane potential can be detected by observing changes from red to green fluorescence using confocal microscopy after JC-1 labeling (19). Additionally, following the protocols provided in the apoptosis assay kit and the mitochondrial membrane potential assay kit, cellular samples from the 12-well plates were collected, stained, and analyzed for apoptosis rate and changes in mitochondrial membrane potential using flow cytometry (Beckman, USA) and FlowJo software.

### 2.7 Evaluation of baicalin’s impact on SVCV-induced mitochondrial damage

Following the identical treatment of the cells, mitochondrial staining was performed using MitoTracker (Thermo Fisher, USA). Mitochondrial morphology was subsequently observed using confocal microscopy. Intracellular levels of reactive oxygen species (ROS) were assessed using a ROS Assay Kit with Dihydroethidium (DHE) and 2’,7’-dichlorodihydrofluorescein diacetate (DCFH-DA) probes (Beyotime, China). Cells were collected for probe loading, and ROS levels were detected and analyzed via flow cytometry using FlowJo software. Additionally, mitochondrial and cytoplasmic proteins were isolated from the treated cell samples using a cell mitochondria isolation kit (Beyotime, China). The levels of cytochrome c (Cyt-c) protein in the mitochondrial and cytoplasmic fractions were then individually quantified.

### 2.8 Quantitative phosphoproteomic analysis without labeling

EPC cells were cultured in T25 flasks for 1624 h, followed by a 2 h exposure to a virus dilution containing 1×10^3^ TCID_50_/mL. After viral exposure, the medium was replaced with one supplemented with 63 mg/L baicalin, and the cells were incubated for an additional 24 h. Cells were then harvested for protein extraction. Each sample was lysed with 1 mL of buffer containing 20 mM Tris-HCl (pH 7.5), 150 mM NaCl (YuanYe, China), 1% n-Dodecyl-β-D-Maltopyranoside (DDM; Sigma-Aldrich, USA), 1× protease inhibitor, and 1× phosphatase inhibitor (both from MCE, USA). Samples were sonicated on ice for 6 cycles (5 s on, 9 s off, 135 W), then incubated on ice for 60 min to ensure complete lysis. The supernatant was collected by centrifugation at 12,000 rpm for 10 min at 4°C. Protein concentrations were determined using a BCA protein assay kit (Beyotime, China). Subsequently, 500 μg of soluble protein was subjected to enzymatic digestion, phosphopeptide enrichment, and LC-MS/MS analysis as described by Xu et al. (20).

### 2.9 Transfection and observation of plasmids

The GFP-LC3 plasmid (Origene, USA) was transfected into EPC cells using polyethylenimine (PEI; MCE, USA) as the transfection reagent. The transfection system consisted of PEI, DNA, and Opti-MEM (Gibco, USA) in a ratio of 1:2.5:100. The prepared transfection mixture was added to EPC cells at 70% confluence, gently mixed, and cultured for an additional 12 h. Following this, the culture medium was replaced, and the cells were incubated for another 48 hours before being subjected to various treatments. The treatments included MOCK, Baicalin, Starvation (serum-free), Starvation + Baicalin (63 mg/L Baicalin in serum-free medium), Rapamycin (50 nM Rapamycin; MCE, USA), and Rapamycin + Baicalin (50 nM Rapamycin + 63 mg/L Baicalin) (21). The cells were then cultured at 25°C for an additional 24 h. In a separate set of treatments, including MOCK, SVCV, Baicalin, SVCV + Baicalin, 3-MA (5 mM 3-methyladenine), and SVCV + 3-MA (5 mM 3-methyladenine; MCE, USA) (5), the cells were cultured at 25°C for an additional 48 hours. Fluorescence changes were observed using confocal microscopy.

### 2.10 Autophagosome staining

After treating the EPC cells with baicalin, the cells were seeded onto 20 mm circular coverslips and subsequently labeled for autophagosomes using the Autophagy Detection Kit (Sigma-Aldrich, USA). This kit employs proprietary fluorescent markers that are specific to autophagosomes, allowing for direct visualization via confocal microscopy. Specifically, 500 μL of the Autophagosome Detection Reagent (1×) was added to each cell sample, followed by incubation at 25°C for 1 h. Post-incubation, the samples were washed three times with 500 μL of Wash Buffer. To stain the cell nuclei, 500 μL of the Acridine Orange Staining Kit (AO; Beyotime, China) was then added, and the samples were incubated at 25°C for an additional 15 minutes. Finally, the stained cells were examined using confocal microscopy.

### 2.11 Antiviral activity of baicalin under different treatment conditions in zebrafish

Prevention: Zebrafish were randomly assigned to four groups: one control group and three experimental groups, with each group consisting of three replicates containing 50 fish per replicate. Baicalin was dissolved in DMSO and incorporated into the experimental feeds using a freeze-drying method to achieve the desired concentrations. The experimental groups were fed with basal feed supplemented with 0, 6.3 mg/kg, or 63 mg/kg of baicalin. After 28 days of feeding, the zebrafish were exposed to the spring viremia of carp virus (SVCV) at a concentration of 1×10^5^ TCID_50_/mL. Each fish was injected with 20 μL of the virus dilution. Over a subsequent 14-day period, the protection rate, viral load, and the expression levels of antiviral immune genes were evaluated.

Treatment: The grouping of zebrafish was consistent with the prevention experiment. The zebrafish in the experimental groups were challenged with SVCV at a concentration of 1×10^5^ TCID_50_/mL. After 20 hpi, the fish were administered 20 μL of a solution containing 0, 6.3 mg/L, or 63 mg/L of baicalin via oral gavage. The protection rate, viral load, and the expression of relevant antiviral genes were monitored and analyzed.

Horizontal transmission: a total of 9 zebrafish were randomly selected as virus donors following SVCV challenge, and 27 zebrafish were selected as non-challenged recipients. The donor group was exposed to SVCV at a concentration of 1×10^5^ TCID_50_/mL. After 24 h, marked recipient fish were introduced into the tanks, and the tank water was replaced with 2 liters of water containing either 63 mg/L of baicalin or an equivalent volume of DMSO. The expression of viral genes was subsequently assessed in the recipient fish.

### 2.12 Antibody

SVCV-G (Professor Xueqin Liu’s research group, Huazhong Agricultural University), LC3B (L7543; Sigma-Aldrich, USA), P62 (66184-1-Ig; Proteintech, China) Beclin-1 (66665-1-Ig; Proteintech, China), mTOR (66888-1-Ig; Proteintech, China), p-mTOR (67778-1-Ig; Proteintech, China), AMPKα1 (A1229; ABclonal, China), p-AMPKα1 (AP0871; ABclonal, China), Cytochrome C (4272; Cell Signaling Technology, USA), GAPDH (2118; Cell Signaling Technology, USA) and Horseradish peroxidase (HRP)-conjugated Goat anti-Rabbit IgG (H+L)/Mouse IgG (H+L) (AS014/AS003, ABclonal, China).

### 2.13 Western blot

EPC cell total protein was extracted using NP40 lysis buffer (Beyotime, China) under ice-cold conditions. The concentration of total protein was determined according to the instructions provided by the BCA assay kit. Separation of total protein was conducted using 12% sodium dodecyl sulfate-polyacrylamide gel electrophoresis (SDS-PAGE; Yeasen, China), followed by transfer onto polyvinylidene fluoride (PVDF; Sigma-Aldrich, USA) membranes (0.22 μm, 0.45 μm). Blocking was carried out with 5% skim milk for 2 h, followed by overnight incubation with primary antibody (1:1000) at 4°C. Subsequently, after Tris-buffered saline tween (TBST; Servicebio, China) washing, membranes were incubated with secondary antibody (1:3000) for 1 h.

### 2.14 Total RNA extraction and RT-qPCR

Total RNA extraction from both cell and zebrafish tissue samples was conducted according to the manufacturer’s instructions for the RNA extraction kit (Yeasen, China). Subsequent quantification of nucleic acid concentration was performed using a NanoDrop spectrophotometer (ThermoFisher, USA). Reverse transcription of RNA into cDNA was accomplished at a concentration of 500 ng/μL using One-Step gDNA Removal and cDNA Synthesis SuperMix (Transgen, China). The application of Universal TaqMan multiplex qPCR master mix (Yeasen, China) with the ABI StepOnePlus Real-Time PCR Detection System (Applied Biosystems, USA) facilitated quantitative real-time PCR (RT-qPCR) analysis. Each sample was tested in triplicate, utilizing *β-actin* gene as an internal control for normalization. Primer sequences are detailed in **Table 1** (22–25). Relative mRNA expression levels were determined using the 2^-ΔΔCt^ method.

**Table 1:** Sequences of primer pairs.

Genes	Primer sequences (from 5’ to 3’)
*β-actin*-F *β-actin*-R	GCTATGTGGCTCTTGACTTCGA CCGTCAGGCAGCTCATAGCT
SVCV-*g*-F SVCV-*g*-R	GCTACATCGCATTCCTTTTGC GCTGAATTACAGGTTGCCATGAT
SVCV-*n*-F SVCV-*n*-R	AACAGCGCGTCTTACATGC CTAAGGCGTAAGCCATCAGC
*ifn-γ-*F *ifn-γ-*R	ATGATTGCGCAACACATGAT ATCTTTCAGGATTCGCAGGA
*rig*-1-F *rig*-1-R	TTGAGGAGCTGCATGAACAC CCGCTTGAATCTCCTCAGAC
*mx*-F *mx*-R	ATAGGAGACCAAAGCTCGGGAAAG ATTCTCCCATGCCACCTATCTTGG

### 2.15 Statistical analysis

Statistical analyses were performed using SPSS 18.0 (SPSS Inc., Chicago, IL, USA) and GraphPad Prism v8.0.1 (GraphPad Software, San Diego, CA, USA). Data are presented as the mean ± standard deviation (SD) from at least three independent experiments. Comparisons among multiple groups were performed using one-way analysis of variance (ANOVA) followed by Tukey’s multiple comparison test. For comparisons between two groups, an unpaired two-tailed Student’s t-test was used where appropriate. Survival curves were analyzed using the Kaplan–Meier method and compared using the log-rank (Mantel–Cox) test. A P value < 0.05 was considered statistically significant. Phosphoproteomic data were analyzed using R software. The enrichment analysis of the Kyoto Encyclopedia of Genes and Genomes (KEGG) database was performed in R. To reduce false-positive results associated with multiple comparisons, P values were adjusted using the Benjamini–Hochberg false discovery rate (FDR) correction where applicable. Histograms were generated using GraphPad Prism v8.0.1.

## 3 Results

### 3.1 Antiviral activity of baicalin *in vitro*

**Figure 1:**
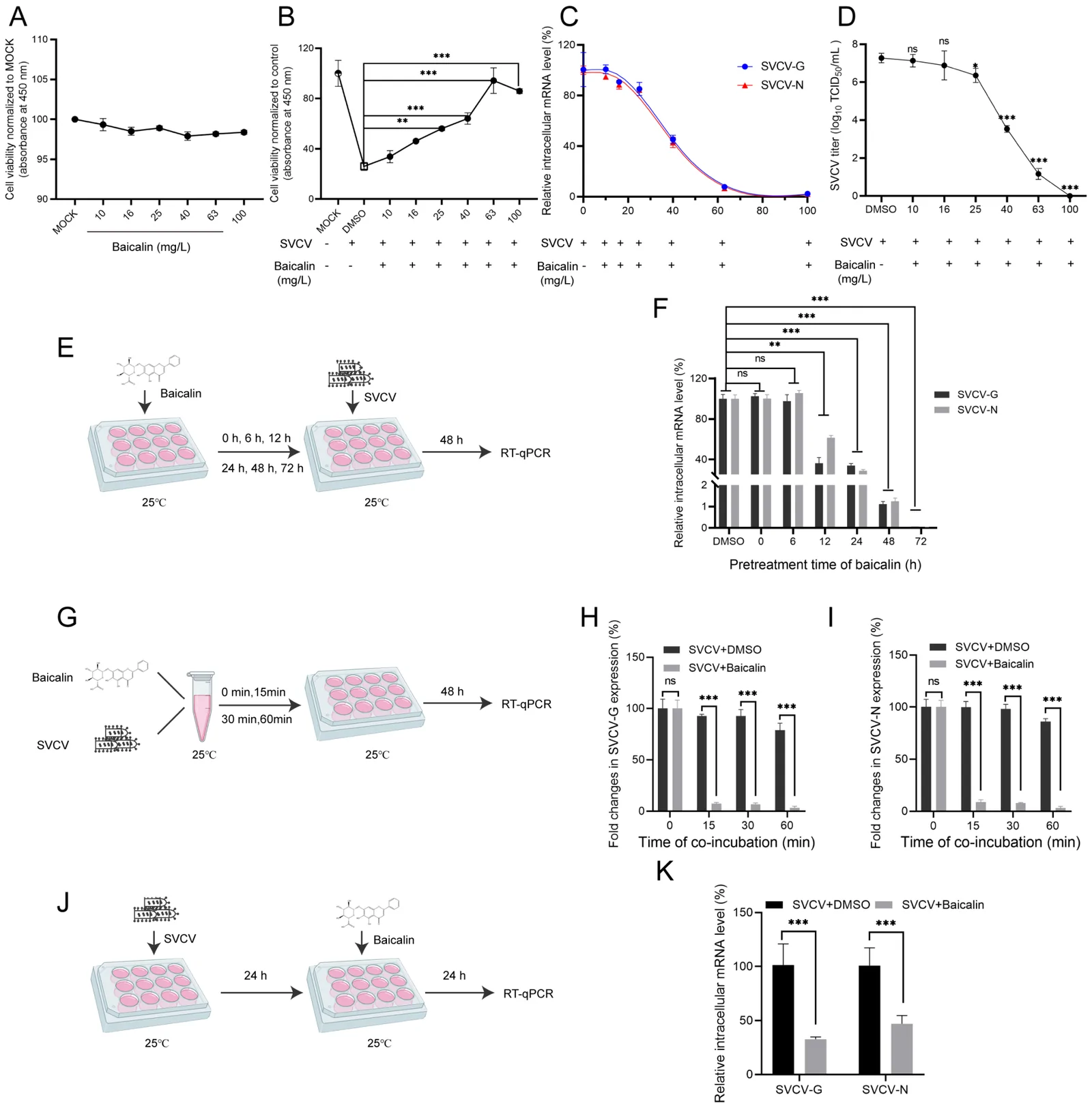
Anti-SVCV effect of baicalin in EPC cells. **(A)** Cytotoxicity of baicalin on EPC cells. Cell viability was detected using the CCK-8 assay after incubation of EPC cells with different concentrations of baicalin for 72 h. **(B)** Effect of baicalin on the viability of SVCV-infected EPC cells. **(C)** Antiviral dose-response curve of baicalin in EPC cells. RT-qPCR analysis was conducted to assess the inhibition rate on the expression of the N and G genes, with normalization against the group of DMSO. **(D)** Viral titers of SVCV-infected and baicalin-treated cells. Viral titers were determined using the Reed-Muench method; **(E, F)** Effect of baicalin pretreatment of EPC cells on SVCV replication. **(G-I)** Effect of co-incubation of baicalin with SVCV on virus particle-infected cells. **(J, K)** Effect of baicalin post-treatment of EPC cells on SVCV replication. Data were shown as mean ± SD (n = 3) from three independent experiments, and significance was analyzed by two-tailed Student’s t-test. (ns, no significant; **p < 0.01; ***p < 0.001).

To determine a non-cytotoxic concentration range and evaluate the antiviral activity of baicalin against SVCV, EPC cells were treated with different concentrations of baicalin prior to cytotoxicity and antiviral assays. CCK-8 data analysis revealed that EPC cell viability remained above 90% after 72 h of exposure to baicalin concentrations ranging from 10 to 100 mg/L (**Figure 1A**). Treatment with baicalin within this concentration range exhibited differential effects on SVCV-infected EPC cells. Notably, a significant reduction in the cytopathic effect (CPE) was observed post-treatment, particularly at 63 mg/L, where cell viability increased to 94.2% (**Figure 1B**). Furthermore, baicalin treatments significantly suppressed viral gene expression, achieving inhibition rates exceeding 95% in both the 63 mg/L and 100 mg/L groups (**Figure 1C**). Additionally, treatment with 100 mg/L baicalin reduced the virus titer to less than 10 TCID_50_/mL, underscoring its potent antiviral efficacy (**Figure 1D**). These findings demonstrate that baicalin effectively inhibits SVCV replication in a dose-dependent manner while exhibiting minimal cytotoxicity toward EPC cells. Based on its optimal balance between antiviral efficacy and cell viability, 63 mg/L was selected as the working concentration for all subsequent mechanistic studies. To further determine the stage(s) of viral infection affected by baicalin, pretreatment, virus–compound co-incubation, and post-infection treatment assays were performed. Baicalin exhibited significant antiviral activity under all three treatment regimens (**Figures 1F–K**), indicating that it possesses prophylactic, direct virucidal, and post-infection antiviral activities.

### 3.2 Baicalin suppresses SVCV-induced apoptosis

**Figure 2:**
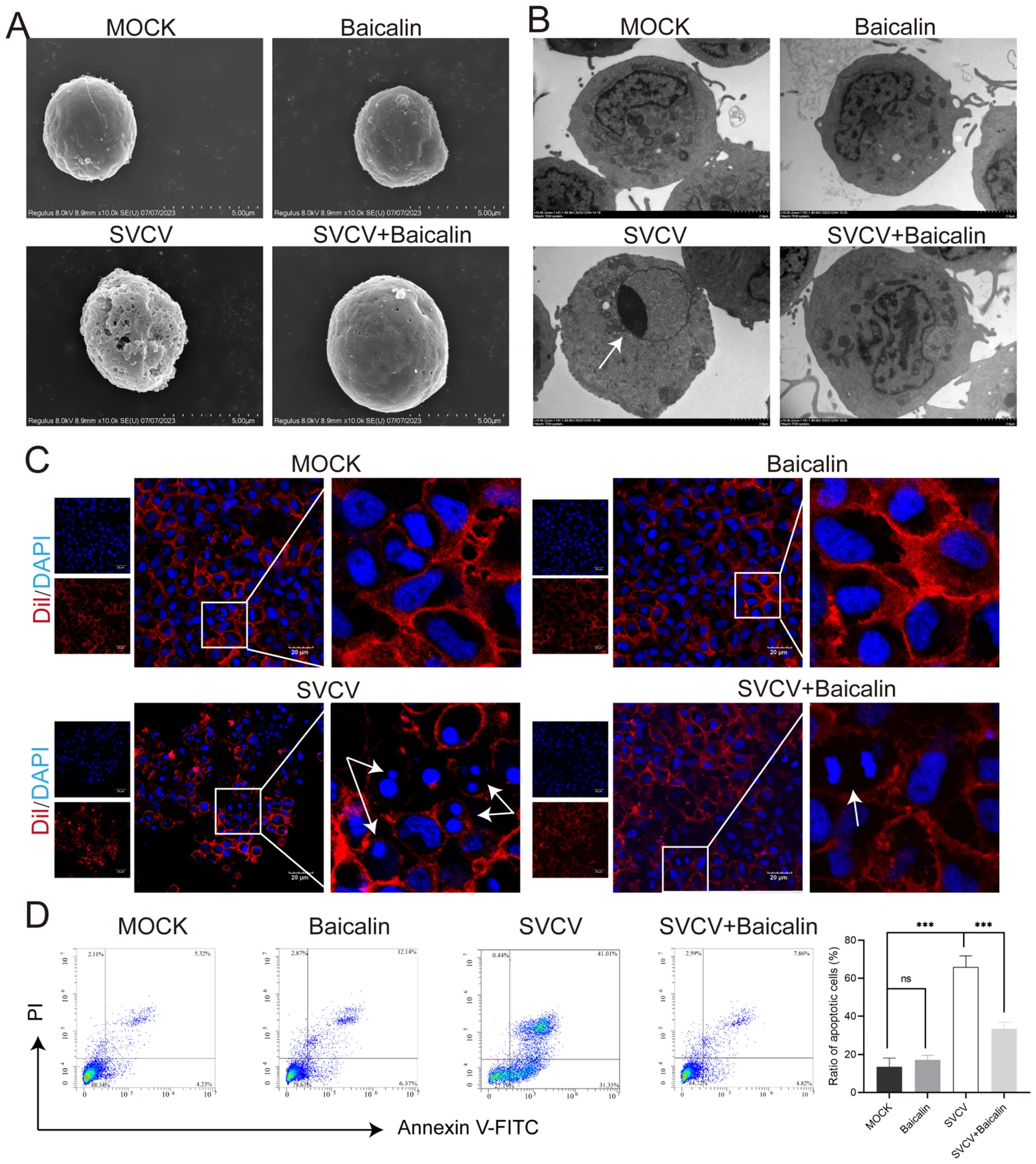
Baicalin suppresses cell apoptosis induced by SVCV. **(A)** Scanning electron microscopy showed the morphological effects of SVCV infection for 48 h on EPC cells and the antiviral activity of 63 mg/L baicalin treatment for 48 h. White arrow: the damaged nuclei. Scale bar: 5 μm, 20 μm. **(B, C)** Observation of cell nuclear damage. White arrow: apoptotic bodies. Scale bar: 20 μm. **(D)** Apoptosis was detected by flow cytometry. Annexin V-FITC and PI were stained, and the proportion of regulated dead cells in each treatment group was detected by flow cytometry. Data are presented as mean ± SD (n = 3) from three independent experiments, and significance was analyzed by two-tailed Student’s t-test. (ns, no significant; ***p < 0.001).

To determine whether baicalin protects EPC cells from SVCV-induced ultrastructural damage, scanning electron microscopy (SEM) and confocal microscopy were performed to evaluate membrane integrity, nuclear morphology, and apoptotic features. SEM results demonstrated that MOCK group cells maintained spherical form. Following infection with SVCV, notable changes occurred: cell shrinkage, irregular, and there was extensive damage to the cellular membrane accompanied by an enlargement of surface gaps, all hallmark signs of apoptosis (**Figure 2A**). SEM and confocal microscopy revealed SVCV-induced nuclear damage and the formation of apoptotic bodies (**Figures 2B, C**; White arrows). SVCV infection induced apoptosis in 65.98% of EPC cells (**Figure 2D**). Baicalin treatment markedly preserved normal cell morphology, maintained plasma membrane integrity, and reduced nuclear damage, resulting in a significant decrease in the apoptotic rate to 33.43%. These findings indicate that baicalin effectively attenuated SVCV-induced apoptosis in EPC cells.

### 3.3 Baicalin ameliorates mitochondrial dysfunction induced by SVCV

**Figure 3:**
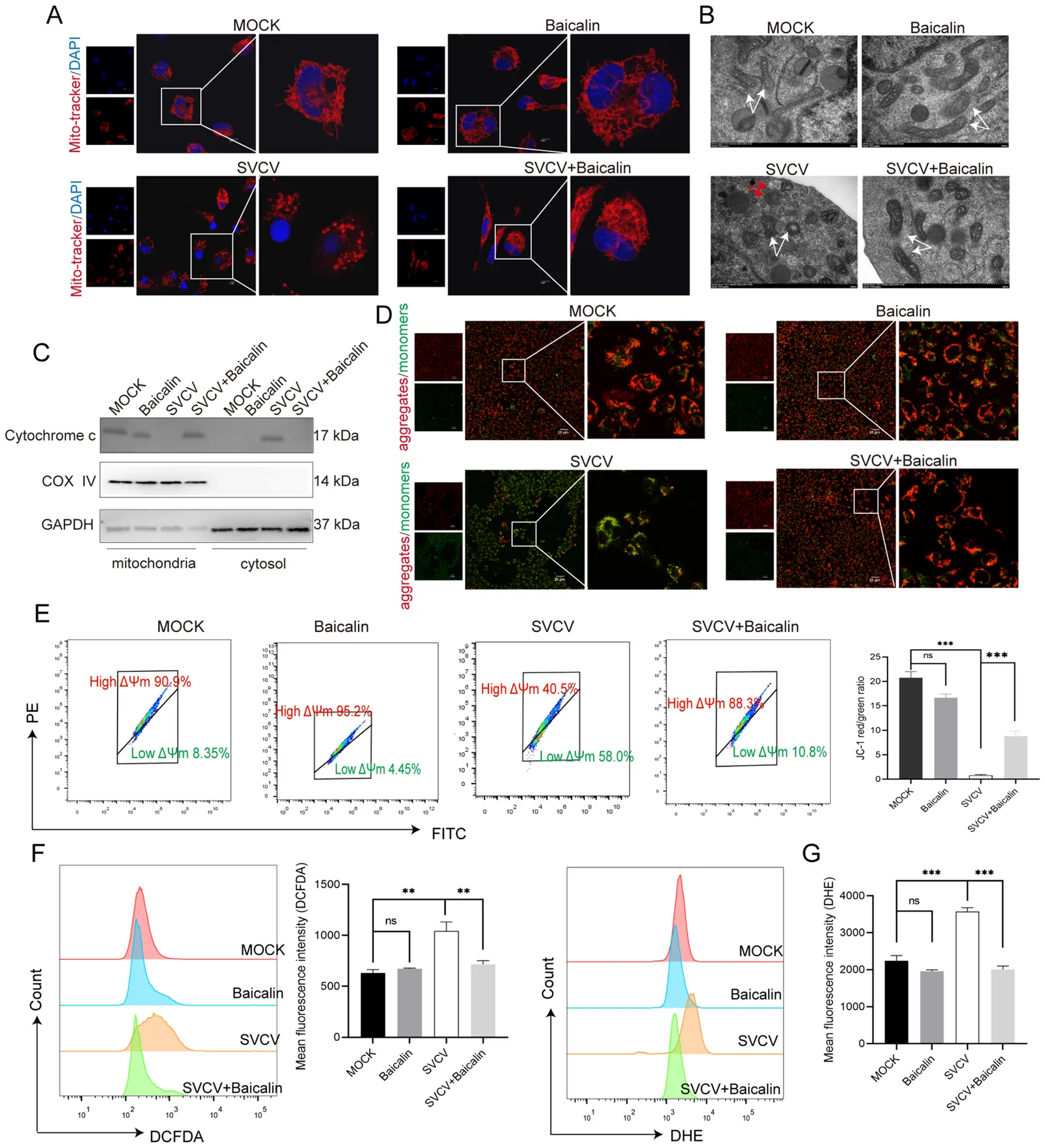
Baicalin alleviates SVCV-induced mitochondrial dysfunction. **(A, B)** Using confocal microscopy and transmission electron microscopy, mitochondrial morphology was observed. SVCV treatment resulted in mitochondrial fragmentation, swelling, and cristae disappearance. Additionally, autophagosomes were observed (red arrows). In contrast, baicalin treatment restored mitochondrial morphology to a more normal state (white arrows). Scale bar: 5 μm. **(C)** Western blot detection of cytochrome C in mitochondria and cytoplasm. Cytochrome C is released from mitochondria into the cytoplasm when mitochondria are dysfunctional. Mitochondria and cytoplasm were separated, and cytochrome C content in mitochondria and cytoplasm was detected using COX IV as the mitochondrial internal reference. **(D, E)** Changes in mitochondrial membrane potential (MMP). JC-1 fluorescent probe was utilized, where mitochondria form aggregates exhibiting a red fluorescence when MMP levels are high, while JC-1 converts to its monomeric form and emits green fluorescence when MMP levels are low. Scale bar: 20 μm. **(F, G)** Detection of intracellular ROS levels using the H2DCFDA and DHE probes via flow cytometry. Mean fluorescence intensity (MFI) was also analyzed. Data are presented as mean ± SD (n=3) from three independent experiments, and significance was analyzed by two-tailed Student’s t-test. (ns, no significant; **p < 0.01; ***p < 0.001).

Because mitochondrial dysfunction and oxidative stress are major contributors to SVCV-induced apoptosis, we next investigated whether the anti-apoptotic effect of baicalin was associated with preservation of mitochondrial integrity. Transmission electron microscopy revealed that SVCV infection caused severe mitochondrial damage, characterized by fragmentation, swelling, loss of mitochondrial cristae, disruption of the mitochondrial network, and a marked reduction in mitochondrial membrane potential (**Figures 3A, B, D, E**), consistent with previous reports by Zhao et al. (3). SVCV infection led to significant mitochondrial damage characterized by fragmentation, swelling into a spherical shape, disappearance of mitochondrial cristae, disruption of the mitochondrial network, and a marked reduction in mitochondrial membrane potential (**Figures 3A, B, D, E**). Interestingly, Western blot analysis revealed a marked translocation of cytochrome C from the mitochondria to the cytoplasm, indicating not only the presence of apoptotic processes but also the accumulation of ROS (**Figure 3C**). We further measured intracellular ROS and DHE levels, finding that cells in the SVCV group exhibited significant ROS accumulation and oxidative stress (**Figures 3F, G**). Notably, after treatment with baicalin, the mitochondrial morphology returned to normal. In 47.2% of the cells, the mitochondrial membrane potential was restored, indicating potent antiviral activity. Furthermore, the cytoplasmic cytochrome C levels decreased, alleviating the oxidative stress caused by SVCV-induced mitochondrial dysfunction.

### 3.4 Phosphoproteomics screening of baicalin’s antiviral pathways

**Figure 4:**
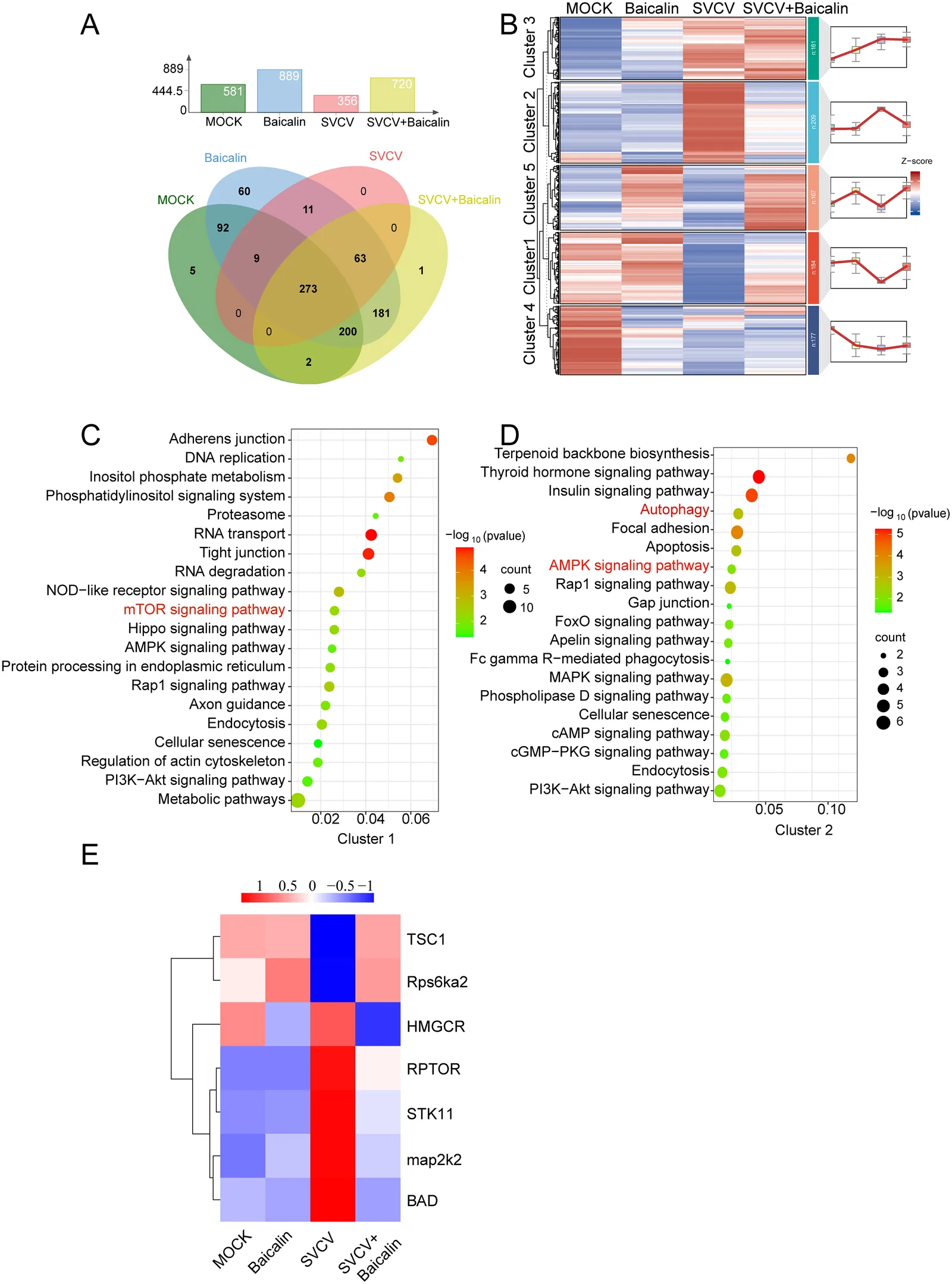
Quantitative label-free phosphoproteomics reveals baicalin antiviral pathway. **(A)** The influence of baicalin on protein phosphorylation modifications. A total of 1254 phosphorylated proteins were identified by mass spectrometry. Among these, 581 phosphorylated proteins were identified in the MOCK group, 889 in the Baicalin group, 356 in the SVCV group, and 720 in the SVCV + Baicalin group. **(B)** Clustering heat map analysis. Based on the filtration with PSM > 1, 898 proteins were selected. These proteins were then categorized into five clusters based on their expression trends across different groups. In Cluster 1, the protein expression decreased in the SVCV group while it increased in the SVCV + Baicalin group. In Cluster 2, the protein expression increased in the SVCV group whereas it decreased in the SVCV + Baicalin group. **(C)** Cluster 1 KEGG analysis. mTOR pathway protein expression was downregulated in the SVCV-treated group and upregulated after Baicalin treatment. **(D)** Cluster 2 KEGG analysis. Autophagy signaling pathway proteins were up-regulated in expression in the SVCV-treated group and down-regulated after baicalin treatment. **(E)** Heatmap of identified mTOR pathway Ampk pathway and autophagy signaling pathway proteins.

To explore the molecular mechanisms underlying the antiviral activity of baicalin and identify the signaling pathways involved, quantitative phosphoproteomic analysis was performed. A total of 1,478 proteins were detected, of which 1,034 were phosphorylated. After filtering for proteins supported by more than one peptide-spectrum match (PSM > 1), 898 phosphoproteins were retained for subsequent analyses (**Figure 4A**). Comparative analysis revealed that SVCV infection markedly reduced the abundance of phosphorylated proteins, whereas baicalin treatment substantially restored protein phosphorylation following viral infection. Specifically, the MOCK, Baicalin, SVCV, and SVCV + Baicalin groups contained 581, 889, 356, and 720 phosphorylated proteins, respectively (**Figure 4A**). Hierarchical clustering further classified the 898 phosphoproteins into five distinct expression patterns (Clusters 1–5) (**Figure 4B**). Among these, Clusters 1 and 2 exhibited the most pronounced responses to SVCV infection and baicalin treatment. Proteins in Cluster 1 were downregulated following SVCV infection but restored by baicalin, whereas proteins in Cluster 2 displayed the opposite expression pattern, suggesting that these proteins may participate in the antiviral mechanism of baicalin. KEGG enrichment analysis identified 153 significantly enriched pathways in Cluster 1 and 164 pathways in Cluster 2 (**Figures 4C, D**). Notably, proteins involved in the mTOR, autophagy, and AMPK signaling pathways were significantly enriched (**Figure 4E**), suggesting that regulation of AMPK/mTOR-mediated autophagy may represent a key mechanism underlying the antiviral activity of baicalin. These findings provided the rationale for the subsequent validation of autophagy regulation.

### 3.5 Baicalin modulates autophagy induced by starvation and rapamycin

**Figure 5:**
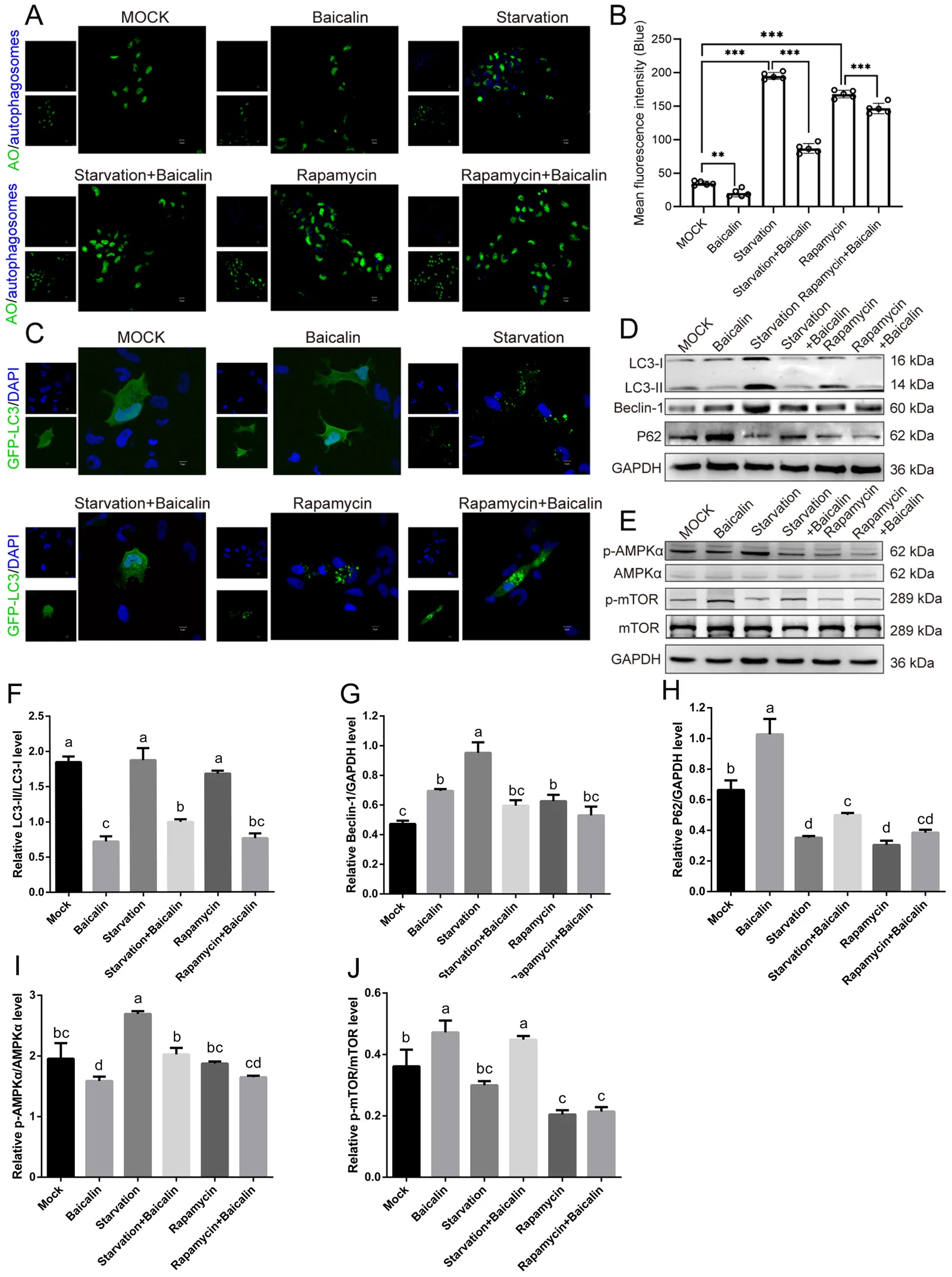
Baicalin modulates starvation- and rapamycin-induced autophagy in EPC cells. **(A, B)** Labeling of autophagosomes (blue) using the Autophagy Detection Kit and counting the average fluorescence intensity of the blue color in five different fields of view. Cell nuclei (Acridine Orange, AO; Green). **(C)** Green fluorescence was observed in EPC cells transfected with GFP-LC3 plasmid, and the green fluorescence was transformed from diffuse to punctate when autophagy occurred. **(D-J)** Western blot to detect the expression levels of autophagy-related proteins (LC3, Beclin-1, P62), p-mTOR, and p-AMPK, where starvation (serum-free) and rapamycin treatment. Data are presented as mean ± SD (n = 3). Different letters indicate statistically significant differences among groups (one-way ANOVA followed by Tukey’s multiple comparison test, P < 0.05).

Since phosphoproteomic analysis indicated that autophagy-related pathways were altered following baicalin treatment, starvation- and rapamycin-induced autophagy models were established to determine whether baicalin directly regulates autophagy independently of SVCV infection. Transmission electron microscopy showed that both starvation and rapamycin markedly increased the number of autophagosomes compared with the control group (**Figures 5A, B**). Consistently, GFP-LC3 fluorescence exhibited a punctate distribution following starvation or rapamycin treatment (**Figure 5C**). Western blot analysis further demonstrated increased LC3-II and Beclin-1 expression accompanied by decreased P62 levels, indicating activation of autophagy (**Figures 5D–J**). Baicalin treatment markedly reduced autophagosome formation induced by starvation, accompanied by decreased GFP-LC3 puncta, reduced LC3-II and Beclin-1 expression, and increased P62 expression. In contrast, baicalin exerted a relatively weaker effect on rapamycin-induced autophagy, although similar trends in autophagy-related protein expression were observed (**Figures 5D–J**).

### 3.6 Baicalin suppresses SVCV-induced autophagy and regulates the AMPK/mTOR signaling pathway

**Figure 6:**
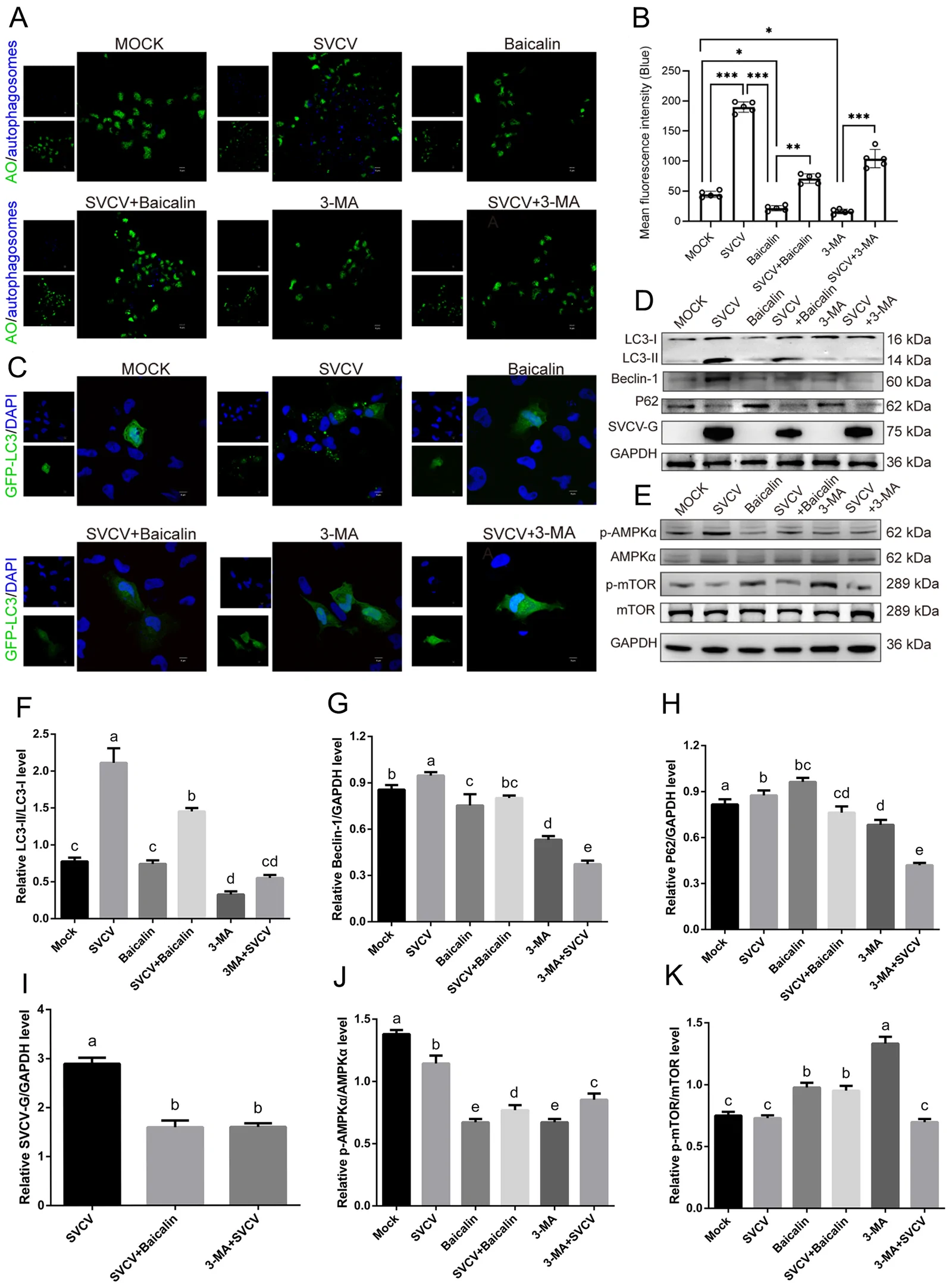
Baicalin suppresses SVCV-associated autophagy and modulates the AMPK/mTOR signaling pathway. **(A, B)** Labeling of autophagosomes (blue) using the Autophagy Detection Kit and counting the average fluorescence intensity of the blue color in five different fields of view. Cell nuclei (Acridine Orange, AO; Green). **(C)** Green fluorescence was observed in EPC cells transfected with GFP-LC3 plasmid, and the green fluorescence was transformed from diffuse to punctate when autophagy occurred. **(D–K)** Western blot to detect the expression levels of autophagy-related proteins (LC3, Beclin-1, P62), SVCV-G, p-mTOR, and p-AMPK. Data are presented as mean ± SD (n = 3). Different letters indicate statistically significant differences among groups (one-way ANOVA followed by Tukey’s multiple comparison test, P < 0.05).

To further determine whether the antiviral activity of baicalin during SVCV infection was associated with suppression of autophagy, and to validate the phosphoproteomic findings, EPC cells were treated with either the autophagy inhibitor 3-MA or baicalin following viral infection. Similar to 3-MA treatment, baicalin reduced SVCV-induced autophagosome formation and GFP-LC3 puncta (**Figures 6A–C**). Western blot analysis showed that baicalin decreased LC3-II and Beclin-1 expression while increasing P62 levels in SVCV-infected cells (**Figures 6D, F-I**), suggesting suppression of SVCV-associated autophagy.

We next examined the AMPK/mTOR signaling pathway. SVCV infection increased AMPK phosphorylation and decreased mTOR phosphorylation, whereas baicalin treatment was associated with reduced AMPK phosphorylation and partially restored mTOR phosphorylation (**Figures 6E, J, K**). These observations indicate that modulation of the AMPK/mTOR-autophagy pathway is associated with the antiviral activity of baicalin against SVCV.

### 3.7 Antiviral activity of baicalin *in vivo*

**Figure 7:**
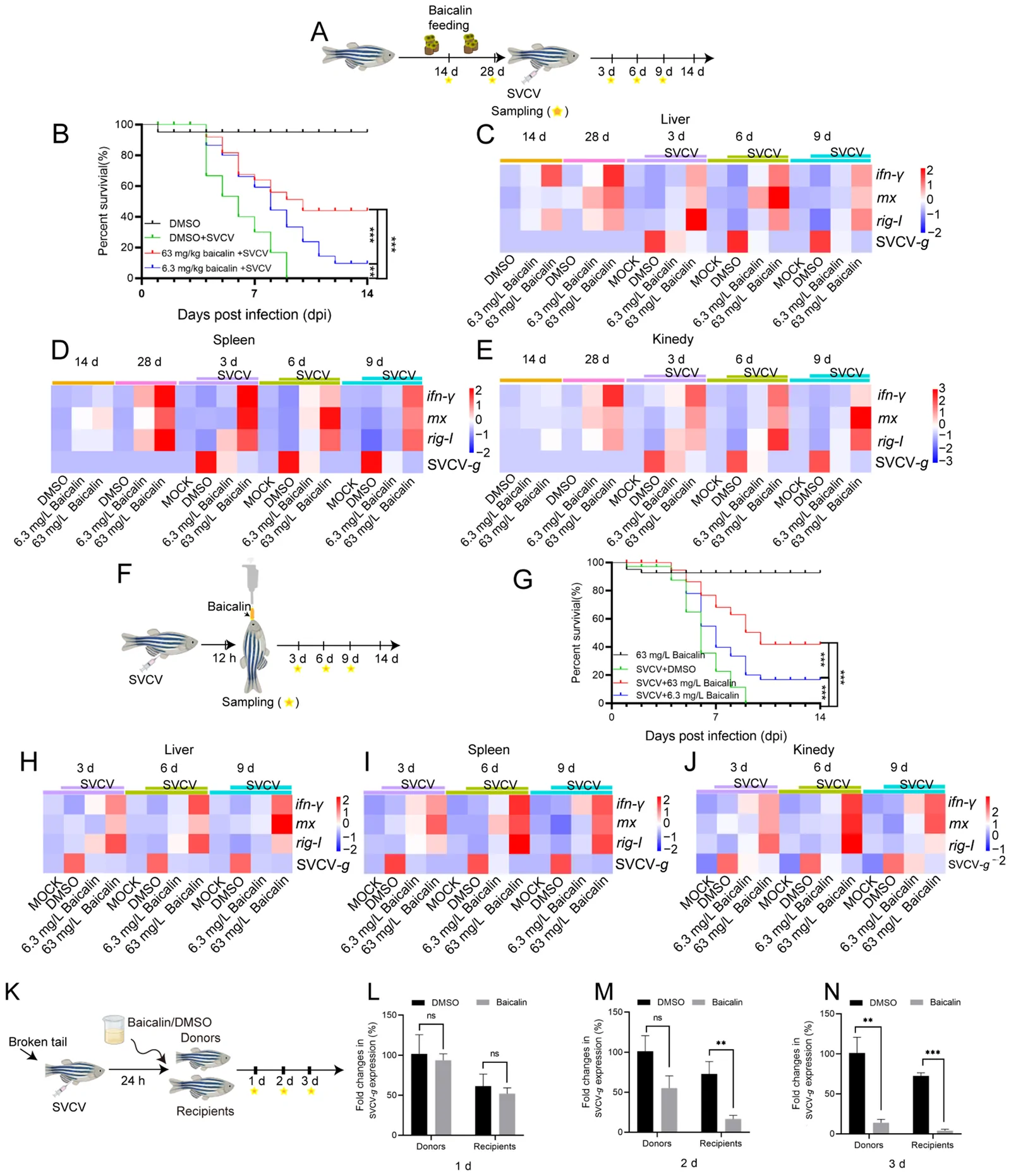
Anti-SVCV effect of baicalin in zebrafish. **(A)** Flowchart of baicalin feeding, viral infection and sampling. **(B)** protection rate following baicalin prophylaxis. **(C–E)** RT-qPCR analysis of the expression of *ifn-γ*, *mx*, and *rig-1* in the liver, spleen, and kidney baicalin fed for 14 d and 28 d and *ifn-γ*, *mx*, and *rig-1* levels at 3 d, 6 d, and 9 d post-viral infection. **(F)** Flowchart of viral infection, baicalin instillation and sampling. **(G)** Protection rate of the therapeutic effect of baicalin. **(H–J)** RT-qPCR analysis of the expression of *ifn-γ*, *mx*, *rig-1*, and SVCV-*g* mRNA levels in the liver, spleen, and kidney at 3 d, 6 d, and 9 d after perfusion feeding. **(K)** Flowchart of the effect of baicalin on the spread of SVCV levels; **(L–N)** RT-qPCR analysis of SVCV-*g* gene expression in tissues of donors and recipients fish at 1 d, 2 d, and 3 d. Data are presented as mean ± SD (n=3) from three independent experiments, and significance was analyzed by two-tailed Student’s t-test. (ns, no significant; **p < 0.01; ***p < 0.001).

To determine whether the antiviral activity of baicalin observed in EPC cells could be translated into *in vivo* protection, zebrafish were used to evaluate its prophylactic, therapeutic, and transmission-blocking efficacy following SVCV challenge. The antiviral efficacy of baicalin in zebrafish was evaluated under both prophylactic and therapeutic regimens. The results demonstrated that both oral (**Figures 7A–E**) and gavage (**Figures 7F–J**) administrations of baicalin significantly upregulated immune-related genes, including *ifn-γ*, *mx*, and *rig-1*, while concurrently downregulating the expression of the SVCV-G gene. These interventions led to increased survival rates in zebrafish, with 43.9% for prophylactic (**Figure 7B**) and 39.7% for therapeutic (**Figure 7G**) applications. Notably, in horizontal transmission experiments, both donor and recipient fish treated with baicalin exhibited a significant reduction in viral load (**Figures 7K–N**). This underscores baicalin’s ability to mitigate the horizontal spread of SVCV and highlights its considerable therapeutic potential in immersion treatments for SVCV-infected fish.

## 4 Discussion

Traditional Chinese medicines and their bioactive constituents have attracted increasing attention as potential antiviral agents because of their broad biological activities and relatively favorable safety profiles (26). Baicalin, one of the major flavonoids isolated from *Scutellaria baicalensis*, has been reported to exhibit antiviral activity against a variety of DNA and RNA viruses, including infectious bronchitis virus, and tilapia lake virus (TiLV) (27, 28). In the present study, baicalin showed no apparent cytotoxicity toward EPC cells within the tested concentration range and significantly inhibited SVCV replication, as demonstrated by improved cell viability, reduced viral titers, decreased viral protein expression, and increased survival of SVCV-infected zebrafish. Collectively, these findings indicate that baicalin possesses significant anti-SVCV activity in both cellular and zebrafish infection models. In the present study, the *in vitro* mechanistic experiments were performed using a viral inoculum of 1 × 10³ TCID_50_/mL, which consistently induced pronounced cytopathic effects while preserving sufficient viable cells for mechanistic analyses, including phosphoproteomics, mitochondrial function, and autophagy. Importantly, the pathogenicity of this viral challenge was further supported by the zebrafish infection model, in which mortality began at 4 days post-infection and reached 100% in untreated infected fish by day 9. Nevertheless, future studies should further evaluate the antiviral efficacy of baicalin under higher viral challenge doses to comprehensively define its therapeutic potential.

Previous studies have shown that SVCV infection induces mitochondrial dysfunction, excessive reactive oxygen species (ROS) production, and apoptosis during the late stage of infection, thereby facilitating viral dissemination and tissue injury (2, 25, 29). Consistent with these observations, our phosphoproteomic analysis revealed significant enrichment of apoptosis-related pathways, including altered phosphorylation of proteins involved in mitochondrial apoptotic signaling. In addition, transcriptomic analysis indicated upregulation of the pro-apoptotic gene *Bad*, suggesting that apoptosis may represent an important host response during SVCV infection. These findings prompted us to further investigate apoptosis-associated events in EPC cells. And our results demonstrated that SVCV infection caused mitochondrial swelling, decreased mitochondrial membrane potential, intracellular ROS accumulation, cytochrome c release, and increased apoptosis in EPC cells. Baicalin markedly alleviated these pathological changes, indicating that maintenance of mitochondrial integrity and redox homeostasis contributes to its cytoprotective effects. Because mitochondrial dysfunction, oxidative stress, and apoptosis are closely interconnected processes during viral infection, the suppression of these cellular injuries may partially account for the reduced viral replication observed following baicalin treatment. These findings suggest that modulation of the host oxidative stress response is likely to contribute to the antiviral activity of baicalin, although the underlying antioxidant defense mechanisms require further investigation. Although apoptosis was not the primary focus of the present study, our findings suggest that baicalin alleviates SVCV-induced mitochondrial apoptosis by reducing oxidative stress and preserving mitochondrial integrity. Because apoptosis and autophagy are closely interconnected during viral infection, suppression of apoptosis may also contribute to the overall antiviral activity of baicalin. However, the precise contribution of apoptosis to viral inhibition requires further mechanistic investigation.

A major objective of this study was to investigate whether autophagy-related signaling is involved in the antiviral activity of baicalin. Interestingly, accumulating evidence indicates extensive crosstalk between autophagy and apoptosis during viral infection. Excessive oxidative stress may simultaneously activate mitochondrial apoptosis while inducing autophagy through AMPK/mTOR signaling, and these two pathways coordinately influence viral replication and host cell survival. Phosphoproteomic analysis identified the AMPK/mTOR signaling pathway as one of the most significantly altered pathways following baicalin treatment. This observation was further supported by Western blot analyses showing modulation of AMPK phosphorylation, mTOR phosphorylation, and the expression of autophagy-related proteins, including LC3-II, Beclin-1, and P62. Furthermore, pharmacological regulation of autophagy using rapamycin and 3-MA produced changes that were consistent with the observed antiviral effects, supporting an association between autophagy-related signaling and SVCV infection.

Autophagy is a self-degradative process that plays a crucial role in balancing energy sources and responding to nutrient stress during critical developmental stages (30). It plays a key role in removing misfolded or aggregated proteins, clearing damaged organelles such as mitochondria, endoplasmic reticulum, and peroxisomes, and eliminating intracellular pathogens (31). Previous studies have demonstrated that SVCV induces endoplasmic reticulum stress and activates autophagy in EPC cells, thereby promoting viral replication (32). Similar proviral functions of autophagy have also been reported for several other aquatic animal viruses. Our phosphoproteomic and biochemical data are consistent with these previous observations and suggest that SVCV infection is accompanied by activation of AMPK/mTOR-associated autophagy, whereas baicalin treatment is associated with suppression of this signaling pathway and reduced viral replication. However, the present study demonstrates an association rather than a direct causal relationship. Because no genetic manipulation of essential autophagy regulators (such as *atg5* or *atg7*) or pathway-specific rescue experiments was performed, our findings do not establish that autophagy is indispensable for SVCV replication or that modulation of autophagy is the direct antiviral mechanism of baicalin. Future studies combining gene knockdown or knockout strategies with pharmacological rescue experiments will be required to determine the causal contribution of autophagy to SVCV infection.

The *in vivo* experiments further demonstrated that baicalin significantly improved the survival of SVCV-infected zebrafish, supporting its antiviral potential under physiological conditions. Zebrafish was selected because it is a well-established laboratory model for investigating SVCV infection, antiviral immunity, and antiviral compound screening, with conserved innate immune responses and high susceptibility to SVCV (33). Nevertheless, zebrafish is primarily an experimental model rather than a major aquaculture species. Moreover, viral burden was evaluated using whole-body samples, and histopathological analyses of major target organs were not performed. Therefore, the present study cannot directly demonstrate tissue-specific inhibition of viral replication or protection against organ damage. In addition, the pretreatment, co-incubation, and post-treatment experiments were not designed to distinguish viral attachment from internalization. Consequently, the precise stage of viral entry affected by baicalin remains to be determined. Future studies should quantify viral loads in major target organs, perform histopathological examinations, employ classical virus attachment and entry assays, and validate the antiviral efficacy of baicalin in economically important cyprinid species such as common carp. Another limitation of this study is that commercially available antibodies raised against mammalian proteins were used for immunoblotting in EPC cells. Although these antibodies detected bands at the expected molecular weights and their expression patterns were consistent with phosphoproteomic and functional analyses, additional validation of antibody specificity in fish cells (e.g., using gene knockdown or knockout approaches) would further strengthen the interpretation of the Western blot results.

## 5 Conclusion

Overall, the present study demonstrates that baicalin exhibits significant antiviral activity against SVCV both *in vitro* and *in vivo*. Our results suggest that the antiviral activity of baicalin is associated with modulation of the AMPK/mTOR-autophagy signaling pathway together with alleviation of mitochondrial dysfunction and apoptosis. Although additional mechanistic studies are required to establish causality, these findings provide new insights into the antiviral properties of baicalin and offer a foundation for further evaluation of this natural compound as a candidate antiviral agent for the prevention and control of viral diseases in aquaculture. Practical application will also require future assessment of formulation stability, delivery strategies, dosage optimization, and environmental safety under aquaculture conditions.

## Data Availability

The raw data supporting the conclusions of this article will be made available by the authors, without undue reservation, to any qualified researcher.
